# Staphylococcal Enterotoxin O Exhibits Cell Cycle Modulating Activity

**DOI:** 10.3389/fmicb.2016.00441

**Published:** 2016-04-15

**Authors:** Elisabeth Hodille, Ludmila Alekseeva, Nadia Berkova, Asma Serrier, Cedric Badiou, Benoit Gilquin, Virginie Brun, François Vandenesch, David S. Terman, Gerard Lina

**Affiliations:** ^1^International Center for Infectiology ResearchLyon, France; ^2^CNRS UMR5308, Inserm U1111, Ecole Normale Supérieure de Lyon – Université Lyon 1Lyon, France; ^3^Institut des Agents Infectieux, Hospices Civils de LyonLyon, France; ^4^UMR1253 STLO, Agrocampus Ouest, Institut National de la Recherche AgronomiqueRennes, France; ^5^Shemyakin-Ovchinnikov Institute of Bioorganic ChemistryMoscow, Russia; ^6^iRTSV-BGE, Université Grenoble AlpesGrenoble, France; ^7^CEA, iRTSV-BGEGrenoble, France; ^8^Biologie à Grande Echelle, Institut National de la Santé et de la Recherche MédicaleGrenoble, France; ^9^Jenomic Research Institute, CarmelCA, USA

**Keywords:** cell cycle alteration, *Staphylococcus aureus*, enterotoxin O, G0/G1 phase delay, cullin-3, cyclomodulin

## Abstract

Maintenance of an intact epithelial barrier constitutes a pivotal defense mechanism against infections. *Staphylococcus aureus* is a versatile pathogen that produces multiple factors including exotoxins that promote tissue alterations. The aim of the present study is to investigate the cytopathic effect of staphylococcal exotoxins SEA, SEG, SEI, SElM, SElN and SElO on the cell cycle of various human cell lines. Among all tested exotoxins only SEIO inhibited the proliferation of a broad panel of human tumor cell lines *in vitro*. Evaluation of a LDH release and a DNA fragmentation of host cells exposed to SEIO revealed that the toxin does not induce necrosis or apoptosis. Analysis of the DNA content of tumor cells synchronized by serum starvation after exposure to SEIO showed G0/G1 cell cycle delay. The cell cycle modulating feature of SEIO was confirmed by the flow cytometry analysis of synchronized cells exposed to supernatants of isogenic *S. aureus* strains wherein only supernatant of the SElO producing strain induced G0/G1 phase delay. The results of yeast-two-hybrid analysis indicated that SEIO’s potential partner is cullin-3, involved in the transition from G1 to S phase. In conclusion, we provide evidence that SEIO inhibits cell proliferation without inducing cell death, by delaying host cell entry into the G0/G1 phase of the cell cycle. We speculate that this unique cell cycle modulating feature allows SEIO producing bacteria to gain advantage by arresting the cell cycle of target cells as part of a broader invasive strategy.

## Introduction

A broad spectrum of exotoxins are produced by *Staphylococcus aureus* that include staphylococcal enterotoxins (SE) and staphylococcal enterotoxin-like toxins (SEl). SEl designates enterotoxins that either lack or have not been tested for emetic properties (Lina et al., 2004). Twenty three such toxins are now recognized designated SE or SEl A to X (Spaulding et al., 2013). These toxins share superantigenic properties by using very low concentrations to bind to the MHCII receptors and activate a large population of T cells via specific vβ regions of the T-cell receptor (TCR) (Marrack and Kappler, 1990). Such polyclonal T-cell mitogenesis results in differentiation into cytotoxic effector cells together with massive secretion of cytokines such as interleukin-2 (IL)-2, interferon gamma (IFN-γ), tumor necrosis factor alpha (TNF-α), and nitric oxide (NO). Several members of this group have been implicated in the pathogenesis of toxic shock syndrome and food poisoning and have shown anti-tumor activity in animal models (Terman et al., 2006).

The most frequently encountered group of SEs are encoded by the enterotoxin gene cluster (egcSEs), an operon consisting of five genetically linked SEs, SEG, SEI, SElM, SElN and SElO and two pseudotoxins (Supplementary Figure S1). These egcSEs alone or together with classic SEs have been identified in up to 80% of *S. aureus* isolates (Jarraud et al., 2001; Becker et al., 2003). While the egcSEs are structurally homologous and phylogenetically related to classic SEA-E, each one exhibits a unique vβ signature (Thomas et al., 2009). egcSEs have been shown to be transcribed in humans during nasal colonization (Burian et al., 2012). Notably, bacteremia with *S. aureus* strains producing egcSEs is reported to be less severe clinically than that linked to *S. aureus* strains producing the classic SEs (Ferry et al., 2005; van Belkum et al., 2006). Despite their broad distribution and occurrence, neutralizing antibodies in human sera directed against the egcSEs are significantly lower than those specific for the classic SEs (Holtfreter et al., 2004).

In a recent clinical study of patients with advanced non-small cell lung cancer, a preparation from a partially purified supernatant from a strain producing only egcSEs induced objective anti-tumor responses (Ren et al., 2004). In search of the mechanisms for the tumoricidal activity of the wild type egcSEs, we demonstrated that egcSEs induce potent NO and TH-1 cytokine dependent tumor killing of a panel of human tumor cells comparable to canonical SEA (Terman et al., 2013).

Superantigens use several mechanisms to induce tumor cell cytotoxicity *in vitro* and *in vivo.* In superantigen dependent cellular cytotoxicity (SDCC) SAgs efficiently bind MHC class II-positive tumor cells which then initiate human T cell proliferation and differentiation into cytotoxic T cells that lyse tumor cells in a perforin/granzyme dependent manner (Dohlsten et al., 1995). MHCII deficient tumor cells can be activated by selected superantigens to express CD154 which costimulates T cell proliferation in a vβ specific manner (Lamphear et al., 1998). Under such conditions T cell activation may be augmented by a recently discovered B7 domain present in selected SEs which interacts with T cell costimulatory receptor CD28 (Arad et al., 2011). Furthermore, both canonical and egc SE-activated T cells and monocytes produce various cytolytic cytokines notably IFN-γ, TNF-α, IL-2 which alone or together with nitrous oxide can induce cytotoxicity in both MHCII+ and MHCII- tumor cells (Fast et al., 1991; Dohlsten et al., 1993). Superantigens have also been shown to activate epithelial cells to produce a broad array of cytokines and chemokines (Peterson et al., 2005). Despite extensive investigation of SAg-cell interactions, classic and egcSEs have not been shown to exert a direct cytostatic effect on target cells.

Here, we further examine the interaction of egcSEs with target tumor cells and unveil a novel property of SEIO, namely the induction of cytostasis in several human tumor cell lines by S phase inhibition during cell cycle progression. Such cytostasis is the result of direct interaction of SEIO with the target cells independent of T cells or TH-1 cytokines. Deploying double hybrid analysis we have also identified cullin-3, a E3 ubiquitin ligase involved in transition from G1 to S phase, as the putative target of SElO. This cell cycle modulating feature constitutes a new cytopathic mechanism by which SEIO alone or together with other SEs can disable host anti-microbial defenses.

## Materials and Methods

### Preparation of Recombinant SEs

SEA, SEG, SEI, SElM, SElN, and SElO were produced in RN6390 *S. aureus* strain transformed with pLUG345 (Boisset et al., 2007) as His-tagged recombinant toxins as previously described for SEA, SEG, SEI, SElM, and SElO (Thomas et al., 2009). We deployed the same strategy to produce SElN using the couple of primers (GAAGGCCTAG ATTGTTCTAC ATAGCTGCAA TTATAATAAC with the addition of a StuI restriction site (underlined) and CCGCGGATCCG TTATTAATGA TGATGATGAT GATGAGAACC CCCATCTTTA TATAAAAATA CATCAATATG ATAATTAG with the addition of a BamHI restriction site) (Thomas et al., 2009). His-tagged recombinant toxins were purified by affinity chromatography on a nickel affinity column according to the supplier’s instructions (New England Biolabs, Ipswich, MA, USA). Protein purity was verified by SDS-PAGE. LPS was removed from toxin solutions by affinity chromatography with Detoxi-GEL endotoxin Gel^®^ (Pierce, Rockford, IL, USA). The QCL-1000 *Limulus* amebocyte lysate assay^®^ (Cambrex-BioWhittaker, Walkersville, MD, USA) showed that the endotoxin content of the recombinant SSAg solutions was less than 0.005 units/mL. Toxins activities were assessed by measuring CD69 surface expression by T cells upon toxin challenge (data not shown).

### Human Tumor Cell Lines

Laryngeal squamous cell carcinoma cell line Hep-2 and human non-small cell lung adenocarcinoma CRL5800 were obtained from cell library (IFR128, Lyon, France). Osteogenic sarcoma CRL1547, human breast cancer cell line MDA-MB-549, human neuroblastoma cell line SK-N-BE and human melanoma PLA-OD were a gift from Raphael Rousseau (Centre Leon Berard, Lyon, France). The human cervix cancer HeLa cells were obtained from American Type Culture Collection, Manassas, VA, USA. Cell lines were cultured in Dulbecco’s modified Eagle medium (DMEM) (Gibco, Invitrogen Corporation, Cergy Pontoise, France) supplemented with 10% fetal calf serum (FCS) (BioWest, Paris, France), 100 U/mL penicillin and 100 μg/mL streptomycin.

### Cytotoxicity Assays

#### MTT and [^3^H]TdR Assays

3-(4,5-Dimethylthiazol-2-yl)-2,5-diphenyltetrazolium bromide (MTT) cytotoxic assay and [^3^H]TdR assay were performed to investigate the effect of SEs on cell viability (Hmama et al., 1993; Terman et al., 2013). Tumor cells (10^5^ cells/well) were seeded in 96-well plates and incubated during 48 h with different concentration of toxin (0, 1, 10, 15 μg/mL) or cisplatin (3 μg/mL) as positive control. For MTT assay, 10 μL of MTT solution (5 mg/mL) (Invitrogen Corporation, Cergy Pontoise, France) was added to culture wells and plates were incubated for 3 h at 37°C. Supernatant was removed and 100 μL of 0.04 N HCl in isopropanol was added to each well before reading optical density at 540 nm (OD_540_) with an ELISA-Reader (Bio-Rad, Marne la Coquette, France).

Cell viability data are expressed as ratio of OD_540_ of treated cells /untreated cells.

For [^3^H]TdR assay, 5 μ Ci of [^3^H]TdR (1Ci/mM, CEA, Sacaly, France) was added during 48h to each well. After three washes with phosphate buffered saline (PBS) without Ca^2+^ and Mg^2+^, cells were collected on glass fibber filters and [^3^H]TdR incorporation was measured in Beckman scintillation counter. Cell viability data are expressed as the percentage of the mean value obtained for untreated cells.

#### Lactate Dehydrogenase (LDH) Release

Tumor cells (10^5^ cells/well) were seeded in 96-well plates and incubated for 48 h with various concentrations of toxin (0, 1, 15 μg/mL). LDH release by tumor cells was assessed by Lactate Dehydrogenase assay on the ARCHITECT Systems^TM^ (Abbott). The nominal range of this assay is 30–2000 U/mL. Maximum LDH value was determined by addition of 10 X lysis solution (9% Tritron X-100 vol/vol) and percentage of cytotoxicity was calculated using the following formula: percent cytotoxicity = (value from test well – value from untreated well)/(value from the maximum – value from untreated well) X 100.

### DNA Fragmentation Assays

DNA fragmentation of tumor cells was detected by the terminal deoxynucleotidyl transferase dUTP nick end labeling (TUNEL) method implemented with the *In Situ* Cell Death Detection Kit TMR-red (Roche applied-science), according to the manufacturer’s instructions. Briefly, tumor cells (10^5^ cells/well) were seeded in Lab-Tek for 48 h with toxins (0, 1, 10, 25 μg/mL) or staurosporine (1 μM) as positive control. After three washes with PBS without Ca^2+^ and Mg^2+^, cells were fixed with paraformaldehyde (4%) during 30 min at room temperature, washed twice and rendered permeable by a solution of sodium citrate and triton X-100 (0.1%) at +4°C. Labeling reaction was carried out with at 37°C for 1 h terminal deoxynucleotidyl transferase, dUTP-rhodamine. Cells were washed twice and seeded before analysis by fluorescence microscopy with Zeiss Axiovert 135 with Axiocam camera (Zeiss, France).

#### Cells Synchronization and Cell Cycle Analysis

Two synchronization protocols were used in the present study. The serum starvataion method arrests the cells in the G0/G1 phase, while the double-thymidine block arrests the cells at the G1/S border (Nougayrède et al., 2005). For cell synchronization by serum depletion Hep-2 cells were grown in a 25-ml flask up to 30% confluence. After washing with PBS, the cells were incubated for 48 h in DMEM supplemented with 0.5% FCS at 37°C with 5% CO_2_ before been cultured for 48 h in the presence or absence of 25 μg/ml of SElO with DMEM supplemented with 5% FCS at 37°C with 5% CO_2_. Cells were them detached by trypsin-ethylenediaminetetraacetic acid (EDTA) (Gibco), washed by PBS and fixed in 70% ethanol 2 h at +4°C. Cells were then washed with PBS, stained with propidium iodide (PI) in presence of 1 mg/mL of RNAse (Sigma) and were analyzed by the flow cytometery (Becton Dickinson, Le Pont de Claix, France) with Mod-Fit (Verity Software House, USA) and FACSDiva 6.2 (BD Biosciences) as described (Nougayrède et al., 2005). Data were collected from 20,000 cells and analysis was performed with CFlow software.

In order to synchronize the cells at the G1/S border, the commonly used double thymidine block (DTB) protocol was employed as described (Deplanche et al., 2015). Briefly, HeLa cells were grown in a 25-mL flask up to 30% confluence. After washing with PBS, the cells were cultured in DMEM containing 10% of FCS supplemented with 2 mM thymidine (DMEM-T) for 18 h. Thymidine was then removed by washing with PBS and the cells were cultured for 9 h to release cells. The cells were then cultured in DMEM-T for 17 h, followed by DMEM containing 10% of FCS. The detached cells were then combined with adherent cells and fixed in 70% ethanol overnight. Cells were then stained with PI and analyzed with an Accuri C6 flow cytometer (BectonDickinson, Le Pont de Claix, France) as described previously (Deplanche et al., 2015). Data were collected from 20,000 cells, and analysis was performed with CFlowsoftware (Becton Dickonson).

### Preparation of *S. aureus* Supernatants

*Staphylococcus aureus* RN6390 pLUG345 (a strain that does not produce any SE and SEls) and RN6390 pLUG345::selo (a strain that produces only his-tag SElO) were used to examine the effect of SElO on cell cycle. Aliquots from overnight cultures of both *S. aureus* strains on Brain Heart Infusion (BHI) broth at 30°C were diluted (1:50) in DMEM and incubated at 30°C for 24 h. The culture supernatants of RN6390 pLUG345 and RN6390 pLUG345::selo strains were collected after separation of the bacteria by centrifugation and adjusted to an optical density (λ = 600 nm) of 0.6. The *S. aureus* supernatants were concentrated 10-fold using a speed-vac (SpeedVac Concentrator SVC11 and Refrigerated Condensation Trap Savant) and pH of the concentrated supernatant was adjusted to 7.4. The superatants were then sterilized by filtration through a 0.22-μm filter (Millipore) and stored at -20°C before the analysis. The total protein concentrations of the concentrated supernatants did not differ by more than 10% between samples.

### Toxins Analysis in *S. aureus* Supernatants

Concentrations of Hla, PSM-α1 and PSM-α3 in RN6390 pLUG345 and RN6390 pLUG::selo preparation were determined by ELISA and HPLC-MS as previously described (Otto et al., 2013; Deplanche et al., 2015). Quantification of SElO in *S. aureus* supernatants was performed using targeted mass spectrometry analysis. Briefly, strain supernatants (100 μL) were reduced for 45 min at 56°C with DTT (10 mM final) and alkylated for 45 min at room temperature in the dark with iodoacetamide (55 mM final). Urea was added (2 M final) to the supernatants before digestion using LysC/trypsin mix (Promega, Charbonnières les Bains, France) at an enzyme/protein ratio of 1/20 (w/w) for 2 h at 37°C. Samples were diluted with 25 mM ammonium bicarbonate to reduce urea to 0.2 M final concentration before performing digestion overnight at 37°C. [^13^C_6_]-lysine labeled peptide (TVDIYGVYYK, HeavyPeptide AQUA Ultimate, Thermo Fisher Scientific, Courtaboeuf, France) was added to the digested samples at a final concentration of 50 pmoles/mL. Then, samples were desalted on C18 columnTip (Proteabio, Morgantown, WV, USA) before drying by vacuum centrifugation. Digests were resolubilized in 40 μL of 2% acetonitrile, 0.1% formic acid and 6 μL were injected on the LC-system. Targeted proteomics analyses were performed on a 6500 QTrap mass spectrometer (AB Sciex, Les Ulis, France) operating in the selected reaction mode (SRM). Liquid chromatography (LC) separation was performed on an ultimate 3000 system (Dionex, Voisins le Bretonneux, France) coupled to a Kinetex XB-C18 column (2.1 × 100 mm, 1.7 μm, 100 Å; Phenomenex, Le Pecq, France). Peptides were separated using a linear 4–40% acetonitrile gradient over 35 min at a flow rate of 50 μL/min. Digested recombinant SElO was used to determine SElO signature peptides and SRM transitions and to schedule acquisition. SElO quantification in *S. aureus* supernatants was derived from the unlabelled/labeled peak area ratios obtained from 3 transitions of the TVDIYGVYYK signature peptide.

### Exposure of HeLa Cells to Bacterial Supernatants

Three hours after the DTB release, HeLa cells were exposed to concentrated *S. aureus* supernatants for 19 h or 22 h. Concentrated DMEM was used as a control at the same time points. The incubation time was chosen in agreement with the recognized evaluation of the phases of the HeLa cell cycle and the previous experiments (Deplanche et al., 2015). All the experiments were performed three times.

#### Yeast Two-Hybrid Analysis

Yeast two-hybrid screening was performed by Hybrigenics Services, S.A.S., Paris, France (http://www.hybrigenics-services.com). The coding sequence for SElO aa 30-261 was PCR-amplified and cloned into pB27 as a C-terminal fusion to LexA (N-LexA-SEO-C) and into pB66 as a C-terminal fusion to Gal4 DNA-binding domain (N-Gal4-SEO-C). The constructs were checked by sequencing and used as a bait to screen a random-primed Human Breast Tumor Epithelial Cells cDNA library constructed into pP6, pB27, pB66 and pP6 derived from the original pBTM116 (Vojtek and Hollenberg, 1995), pAS2ΔΔ (Fromont-Racine et al., 1997) and pGADGH (Bartel et al., 1993) plasmids, respectively.

For the LexA bait construct, we did not obtain any His+ colonies. For the Gal4 construct, 60 million clones (6-fold the complexity of the library) were screened using the same mating approach with HGX13 (Y187 ade2-101::loxP-kanMX-loxP, mata) and CG1945 (mata) yeast strains. A total of 56 His+ colonies were selected from a medium lacking tryptophan, leucine and histidine. The prey fragments of the positive clones were amplified by PCR and sequenced at their 5′ and 3′ junctions. The resulting sequences were used to identify the corresponding interacting proteins in the GenBank database (NCBI) using a fully automated procedure. A confidence score (PBS, for Predicted Biological Score) was attributed to each interaction as previously described (Formstecher et al., 2005).

### Statistical Analysis

*T*-tests were performed to compare level of MTT and incorporation [^3^H]TdR by cell lines challenged or not by toxins. The differences among the cell cycle phases were assessed by analysis of variance (ANOVA). Tukey’s Honestly Significant Difference test was applied for comparison of means between groups. The level of statistical significance was set at 0.05. The tests were carried out with SPSS Statistics^®^ version 22 software (IBM France, Bois Colombes, France).

## Results

### SElOs Inhibits Proliferation of a Large Number of Tumor Cell Lines But Does Not Induce Cell Death

**FIGURE 1:**
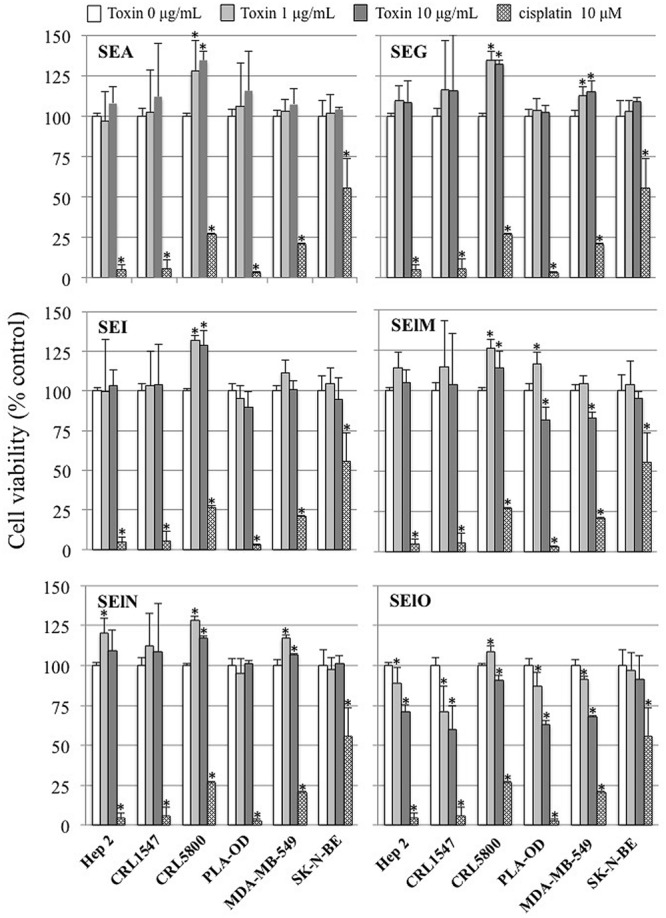
**SElO inhibits cell line viability.** Hep-2, CRL5800, CRL 1447, MDA-MB-549 SK-N-BE and PLA-OD cell lines (10^5^ cells) were incubated with or without SEA, SEG, SEI, SElM, SElN, SElO (1–10 μg/mL) or cisplatin (10 μM) as positive control during 48 h. At the end of this incubation period, cell viability was assessed using the MTT assay. Cell viability data are expressed as 100 X ratio of OD_540_ of treated cells/untreated cells. Values are mean ±SD (*n* = 3 independent experiments for Hep-2, CRL1447 cell lines and one experiment for CRL5800, MDA-MB-549 SK-N-BE and PLA-OD cell lines, each experiment containing 3 replicates). ^∗^*P* < 0.05, vs. negative control without toxin.

**FIGURE 2:**
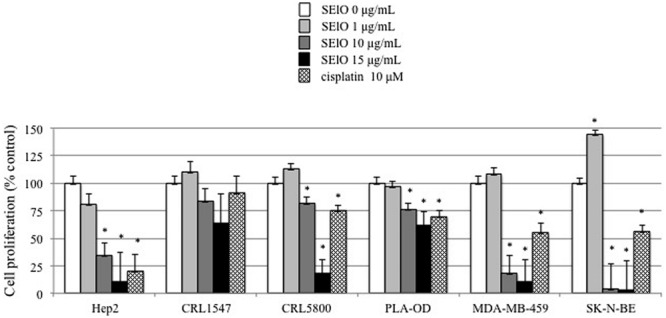
**SElO inhibits cell line proliferation.** Hep-2, CRL5800, CRL 1447, MDA-MB-549 SK-N-BE and PLA-OD cell lines (10^5^ cells) were incubated with or without SElO (0, 1, 10, 15 μg/mL) or cisplatin (10 μM) as positive control during 48 h. At the end of this incubation period, cell viability was assessed using [^3^H]TdR assay for SElO only. Cell viability data are expressed as 100X ratio of value of treated cells/untreated cells. Values are mean ±SD (*n* = 4 independent experiments for Hep-2, CRL1447, CRL5800, MDA-MB-549 and PLA-OD cell lines and *n* = 2 for SK-N-BE, each experiment containing 2 replicates). ^∗^*P* < 0.05, vs. negative control without toxin.

**FIGURE 3:**
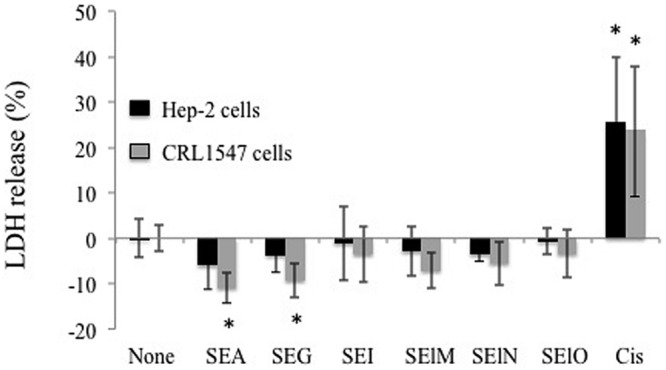
**SElO do not induce cell necrosis.** Hep-2 and CRL5800 were incubated with or without SEA, SEG, SEI, SElM, SElN, SElO (10 μg/mL) or cisplatin (10 μM) as positive control during 48 h. At the end of this incubation period, cell necrosis was assessed by LDH quantification in the supernatant. The results are expressed as percentage of LDH release: % LDH release = (value from test well – value from untreated well)/(value from lysis solution – value from untreated well) X 100. Value and are mean ±SD. (*n* = 3 independent experiments, each experiment containing 3 replicates). ^∗^*P* < 0.05, vs. negative control without toxin.

**FIGURE 4:**
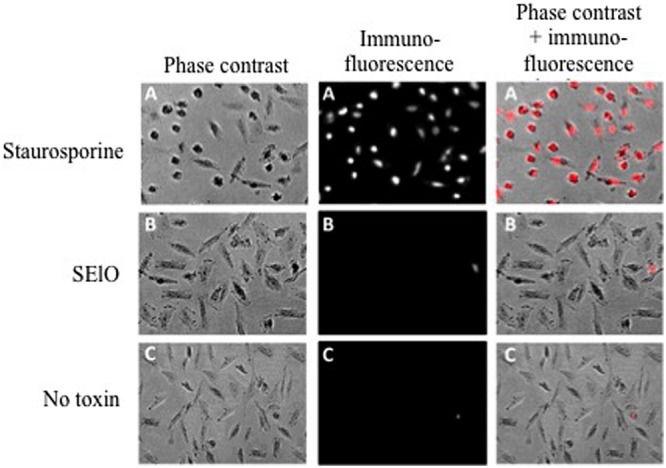
**SElO do not induce cell apoptosis.** Hep-2 cell (10^5^ cells) were incubated in DMEM + 10% FCS for 48 h with 10 μg/mL of SElO **(B)**, 1 μM of staurosporine (positive control, **A**) or no toxin (negative control, **C**). Cells were fixed with paraformaldehyde (4%), permeabilised by a solution of sodium citrate and triton X-100 before DNA 3′ end labeling by dUTP-rhodamine. Cells were seed before analysis by fluorescence microscopy with Zeiss Axiovert 135 with Axiocam camera. Data are from 1 of 3 independent experiments that gave similar results.

We first examined cell viability of 6 cell lines in the presence of SEs using the MTT cytotoxicity assay. SEA, SEG, SEI, and SElN did not significantly alter cell viability of these cell lines. Exposure to SElO surprisingly induced a dose dependent reduction of cell viability (from 60 to 91% with 10 μg/mL), which was statistically significant in 5 of the 6 cell lines (*p* < 0.05) (**Figure 1**). SElM had variable effect at high concentration, inducing proliferation of 1 of the 6 cell lines and inhibition of proliferation in 2 of the 6 cell lines (**Figure 1**). We then examined cell proliferation of 6 cell lines in the presence of SElO by measuring [^3^H]TdR incorporation (**Figure 2**). SElO induced a dose dependent inhibition of proliferation (from 3 to 64% with 15 μg/mL) in 5 of the 6 cell lines tested (*p* < 0.05). Next, we determined whether SEIO’s ability to inhibit tumor cell proliferation could be ascribed to a cytotoxic or cytostatic effect on the tumor cells. Thus, we examined the effect of SEA and the egcSEs on various tumor cell lines in an LDH assay. For these tests, we used CRL-5800 and Hep-2 as target cells because they were reproducibly sensitive to inhibition of proliferation by SElO. LDH values measured in supernatants of tumor cells treated with SElO were no greater than those of the negative controls (LDH released <5%) (**Figure 3**). To determine if SElO might be inducing tumor cell apoptosis we tested the SEIO-exposed cells in a TUNEL assay. Significant DNA fragmentation induced by SElO at the concentrations used in our assay (1–25 μg/mL) relative to staurosporine was not observed (**Figure 4**). The absence of cell apoptosis induction by SElO was confirmed by Annexin-V-FITC/PI staining and caspase 3 measurement (data not shown). Hence, the inhibition of proliferation induced by SEIO could not be ascribed to target cell necrosis or apoptosis.

### SElO Delays the Cell Cycle at the G0/G1 Phase

**FIGURE 5:**
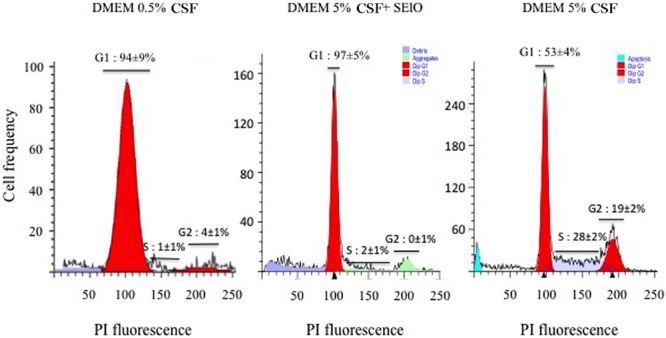
**SElO delays the cell cycle at the G0/G1 phase.** Hep-2 cells (10^5^ cells) were synchronized in G1 by serum depletion in DMEM with 0.5% FCSFCS before being cultured during 48 h in DMEM supplemented with 5% FCS in presence or absence of 25 μg/mL SElO. After fixation and permeabilization, cells were stained by PI. Fluorescence of the PI-stained cells was measured by flow cytometry and Mod-Fit deconvolution software that provided the estimated frequency of cells with fractional DNA (debris, aggregate, apoptotic cells) and cells in G1, S and G2/phases of the cycles.

The inhibition of the host’ cell proliferation by SEIO in the absence of necrosis or apoptosis could be related to the modification of the host cell cycle by SElO. To verify this hypothesis the cell cycle was synchronized by serum starvation. Hep-2 cells exposed to high concentration SElO were compared to untreated synchronized cells. The level of cell synchronization was verified using flow cytometry analysis after 48 h of incubation in serum free media: 94 ± 9% cells were in G0/G1. Analysis of DNA contents indicated that 47 ± 4% of the cells were in S and G2/M phases in the absence of SElO. In contrast, only 2 ± 1% of cells were in S and G2/M phases upon exposure to SEIO for 48 h (**Figure 5**). These data clearly indicate that most of the cells were delayed in the G0/G1 phase by SElO. It is noteworthy that less than 1% of apoptotic cells were detected during the experiments in presence or absence of SElO, confirming that SElO did not induce direct cell apoptosis.

### Delay in G0/G1 Depends on the Production of SEIO

Other *S. aureus* toxins, PSMα1 and PSMα3 and Hla, alter the cell cycle progression and delay G2/M phase transition (Haugwitz et al., 2006; Deplanche et al., 2015). To determine the role of SElO compared to PSM and Hla in the alteration of the host cell cycle by *S. aureus*, we compared the DNA content of synchronized HeLa cells exposed to concentrated supernatants of RN6390 transformed with pLUG345 or pLUG345::*selo*. PSM-α1, PSM-α3 and Hla concentrations were identical in RN6390 pLUG345 and RN6390 pLUG345::selo supernatants, at 0.03 μg/mL, 0.05 μg/mL and 0.05 μg/mL, respectively. By contrast, targeted proteomics analysis demonstrated the presence of SElO in RN6390 pLUG345::selo only, at a concentration of 4.7 μg/mL.

**FIGURE 6:**
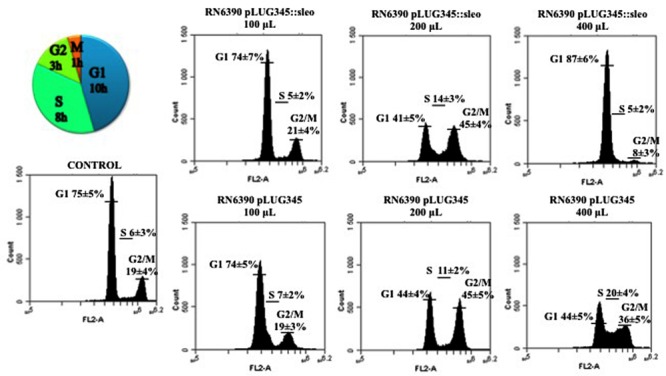
**Supernatant of SElO producing *S. aureus* induces delay in transition of G1 in S phase.** Synchronized HeLa cells were exposed either to 10-fold concentrated supernatant of *S. aureus* RN6390 producing SEIO, RN6390 devoid of SEIO, or DMEM. After 19 h of incubation, detached and adherent cells were fixed in 70% ethanol overnight, stained with PI and analyzed by flow cytometry. Data were collected from 20,000 cells and analysis was performed with CFlow software. The average percentage of cell cycle phase ± SD is indicated. The values of one representative assay out of the four is shown. Exposure of cells to the *S. aureus* wild type supernatant induced a G0/G1 phase transition delay.

After 19 h of exposure of the HeLa cells synchronized by the double block thymidine to 200 μl of concentrated supernatant of either RN6390 pLUG345 or RN6390 pLUG345::selo, a G2/M phase transition delay was detected by cytofluorometry analysis (**Figure 6**). The percentage of cells in the G2/M phase after the treatment with RN6390 pLUG345 and RN6390 pLUG345::selo supernatants was 45 ± 4% and 45 ± 5% respectively compared to 19 ± 4% in control cells. The G2/M phase transition delay corresponds to the effect of PSM-α1, PSM-α3 and Hla, detected in supernatants of both strains.

An increase in the volume of supernatants resulted in G1/S phase delay. Thus, after exposure of HeLa cells to 400 μl of concentrated supernatants of RN6390 pLUG345::selo strain producing SElO, the percentage of cells in the G1 phase was higher (87 ± 6%) compared to untreated cells (75 ± 5%) while only 44 ± 5% of HeLa cells exposed to 400 μl of concentrated supernatants of RN6390 pLUG345 were in the G1 phase (**Figure 6**, **Table 1**). This proportion was stable 3 h later since the number of cells exposed to concentrated supernatants of SElO producing strain in the G1 phase was still higher (89 ± 6, compared to untreated cells (78 ± 5%) of which 41 ± 4% were exposed to concentrated supernatants of the control strain (**Table 1**).

**Table 1:** Impact of SElO production on the effect of *S. aureus* supernatant on the cell cycle.

Experimental conditions	G1	S	G2/M
**Asynchronous cells**	61 ± 5%	17 ± 3%	22 ± 4%
**Synchronous cells**			
Time after DTB release 19 h			
Control	75 ± 5%	6 ± 3%	19 ± 4%
Cells + 100 μL RN6390 pLUG345::sleo	74 ± 7%	5 ± 2%	21 ± 4%
Cells + 200 μL RN6390 pLUG345::sleo	41 ± 5%	14 ± 3%	45 ± 4%
Cells + 400 μL RN6390 pLUG345::sleo	87 ± 6%	5 ± 2%	8 ± 3%
Cells + 100 μL RN6390 pLUG345	74 ± 5%	7 ± 2%	19 ± 3%
Cells + 200 μL RN6390 pLUG345	44 ± 4%	11 ± 2%	45 ± 5%
Cells + 400 μL RN6390 pLUG345	44 ± 5%	20 ± 4%	36 ± 5%
Time after DTB release 21 h			
Control	78 ± 5%	12 ± 3%	10 ± 3%
Cells + 100 μL RN6390 pLUG345::sleo	84 ± 7%	5 ± 3%	11 ± 3%
Cells + 200 μL RN6390 pLUG345::sleo	69 ± 6%	9 ± 3%	22 ± 4%
Cells + 400 μL RN6390 pLUG345::sleo	89 ± 5%	8 ± 2%	3 ± 2%
Cells + 100 μL RN6390 pLUG345	87 ± 8%	6 ± 2%	7 ± 2%
Cells + 200 μL RN6390 pLUG345	85 ± 8%	6 ± 2%	9 ± 3%
Cells + 400 μL RN6390 pLUG345	41 ± 4%	20 ± 4%	39 ± 5%

*After incubation, detached and adherent HeLa cells were fixed in 70% ethanol overnight, stained with PI and analyzed by flow cytometry. Data were collected from 20,000 cells and analysis was performed with CFlow software. The average percentage of cell cycle phase ± SD is indicated.*

### Yeast Two-Hybrid Analysis

We extended our analysis of cell cycle subversion by SElO by performing a yeast-two-hybrid study using a mating assay protocol as described in “Material and Methods.” One construct was made with full length of the coding sequence of the mature SElO fused C-terminal tp Gal4 DNA binding domain and tested against a human breast tumor epithelial cell library as described. Extensive library screening identified two prey with low confidence: a putative protein, homologue of jbug (GID 281376931) and cullin-3 (CUL3) (Supplementary Data 1 and 2). CUL3 3 is a E3 ubiquitin ligase involved in cell cycle regulation through cyclin E, a evolutionarily conserved protein whose essential function is to promote the cell cycle transition from G1 to S phase.

## Discussion

Pathogens have developed sophisticated mechanisms which allow them to hijack host cell functions to their own benefit. There is a growing body of the evidence showing pathogen induced alteration of the host cell cycle which is the major process leading to the cellular proliferation that is required for tissue remodeling (Nougayrède et al., 2005). Few studies have described the capacity of *S. aureus* to alter the host cell cycle. For instance, it was observed that an epidermal cell differentiation inhibitor suppresses keratinocyte differentiation (Sugai et al., 1992) and Hla increases the continuation of S+G2/M phases (Haugwitz et al., 2006) while S. *aureus* toxin PSM alpha induces G2/M transition delay (Deplanche et al., 2015).

As a suitable cellular model for investigation of the effect of *S. aureus* on host cell proliferation, death and cell cycle progression, we chose a broad and diverse group of human tumor cell lines including the epithelial cervix cancer HeLa cells, which are widely used as a model for cell cycle research (Alekseeva et al., 2013; Deplanche et al., 2015). Using recombinant enterotoxins we showed that from a group consisting of SEA and the egcSEs, SEIO reproducibly inhibited proliferation of the 5/6 of the cell lines tested while SElM also inhibited 2/6 cell lines tested. Focusing on SElO, additional experiments excluded cell death (necrosis and apoptosis) in the observed inhibition of the proliferation. Since all toxins were produced individually using the same *S. aureus* strain and purified by the same methods, the observed differences in egcSE cytostatic behavior reflect authentic variation in their functional activity. At the amino acids level, percent of identity of mature toxin tested varies from 28 to 61%; SElO shares only 31 to 40% identity with SEA and other egcSEs, and is phylogenically distant from the other SEs and SEls (Thomas et al., 2009). Likewise, differences in sensitivity of the cell lines to toxins reflect intrinsic variations in complementary receptors and activation pathways yet to be undefined.

The investigation of DNA content in host cells synchronized by serum starvation and exposed to SEIO clearly indicates the arrest of host cells in G1 phase. Such arrest occurs at a different phase of the cell cycle than that observed previously with other *S. aureus* toxins (Haugwitz et al., 2006; Deplanche et al., 2015). The ability of SElO to induce G1 arrest was confirmed by showing that only crude supernatants of SEIO producing isogenic *S. aureus* strains inhibited cell cycle progression of target cells synchronized by the double block thymidine HeLa cells. At lower concentration RN6390 pLUG345 and RN6390 pLUG345::selo strains induced G2/M phase transition delay, evidenced by the presence of PSM-α1, PSM-α3 and Hla in supernatants of both strains.

SEIO’s ability to inhibit cell cycle progress was further corroborated by yeast-two-hybrid studies which indicated that Cul3 is a potential target for SEIO. Cullins are a family of proteins that act as scaffolds conferring substrate specificity to multimeric complexes of E3 ligases. The function of Cul3 in cell cycle regulation is well documented (Andérica-Romero et al., 2013). In addition to the capacity of Cul3 to regulate the entry to mitosis, Cul3 plays a pivotal role in a degradation of cyclin E, an evolutionarily conserved protein whose essential function is to promote the cell cycle transition from G1 to S phase (Knoblich et al., 1994).

The above data provides evidence that staphylococcal enterotoxin SElO delays host cell entry into the G0/G1 phase of the cell cycle. We identified two *S. aureus* toxins PSM alpha (Deplanche et al., 2015) and SElO as cyclomodulins. Various cyclomodulins are described in *Escherichia coli* such as cytotoxic necrotizing factor, the cycle-inhibiting factor, and two kinds of genotoxins, cytolethal distending toxins and colibactin (Nougayrède et al., 2006). We speculate that SElO-producing *S. aureus* may gain advantage in the host by arresting the cell cycle and inducing phenotypic changes that enhance bacterial propagation and invasion.

Comparable to other egcSEs, SElO selectively stimulates T cells via the Vβ region of the T cell receptor (Jarraud et al., 2001; Thomas et al., 2009) and displays an ability to induce apoptosis in a broad panel of human tumor cells in a nitrous oxide and cytokine dependent mechanism manner (Terman et al., 2013). To this growing list of properties we now add SElO’s cytostatic effect induced by cyclomodulin producing target cell alterations that may promote *S. aureus* colonization. We theorize that target tumor cells undergoing cytostasis in response to SElO may be rendered susceptible to the cytotoxic effect of CD8+ T cells or a constellation of tumoricidal nitrites, cytokines and perforin granzyme known to be induced by the egcSEs and classical SEs (Rosendahl et al., 1998). In this context, SElO was identified together with other egcSEs in a staphylococcal supernatant used to successfully treat 14 patients with advanced lung cancer and pleural effusion (Ren et al., 2004; Terman et al., 2006). Notably, several chemotherapeutics and small molecules used in cancer treatment are cell cycle specific (Chabner and Longo, 2011). Likewise, several non-chemotherapeutics and biologics such as Lovastatin, methylxanthine, trifluoperazine, chloropromazine, caffeine, sodium ascorbate and vitamin D induce alterations in cell cycle kinetics of tumor cells and can synergize with other agents to produce tumor cytotoxic effects (Stewart and Evans, 1989). Hence, in addition to its ability to disable host anti-microbial defenses in its natural state, SElO acting alone or together with other SEs or drugs may plausibly be harnessed as an adjunct for cancer treatment.

## Author Contributions

NB, VB, FV, DT, and GL designed the study. EH, LA, AS, CB, BG, CB, and HC performed the experiments. DT, NB, VB, and GL wrote the manuscript. EH and FV edited and modified the manuscript.

## Conflict of Interest Statement

The authors declare that the research was conducted in the absence of any commercial or financial relationships that could be construed as a potential conflict of interest.
