# Supplementary material for: Staphylococcal Enterotoxin O Exhibits Cell Cycle Modulating Activity
Source: Front Microbiol. 2016 Apr 15;7:441. doi: 10.3389/fmicb.2016.00441 (PMC4832122; doi:10.3389/fmicb.2016.00441)
Supplement: Supplementary file 1 [file Data_Sheet_1.PDF]

HYBRiGeNiCS

services

DomSight: HBEC_RP1_hgx2542v1 vs. Human Breast Tumor Epithelial Cells_RP1 (05 Nov 2010)

(Bait plasmid(s): hgx2542v1_pB27, hgx2542v1_pB66)

legend	
bait fragment	SID: Selected Interaction Domain
sid fragment	It is the amino acid sequence shared
interpro domain	by all prey fragments matching the
other domain (pfam, smart, ...)	same reference protein.
transmembrane domain	SIDs have been found in numerous cases
coiled-coil domain	to correspond to an identified structural
signal peptide	or functional domain.

legend

SEO
Staphylococcus aureus

1 261
30 261 hgx2542v1
7 29 Transmembrane domain
1 29 Signal peptide domain
28 151 Enterotoxin, bacterial- INTERPRO
52 146 Staphylococcal/ Streptococcal toxin, OB- f- INTERPRO
101 127 Superantigen, bacterial- INTERPRO
116 261 Superantigen toxin, C- terminal, Staphylo- INTERPRO
135 148 Superantigen, bacterial- INTERPRO
137 149 Toxin, bacterial- INTERPRO
139 148 Staphylococcal enterotoxin/ Streptococcal- INTERPRO
154 260 Toxin, beta- grasp, Staphylococcal/ Strept- INTERPRO
176 192 Toxin, bacterial- INTERPRO
176 203 Superantigen, bacterial- INTERPRO
242 258 Superantigen, bacterial- INTERPRO
28 151 Bact_endotox- SSF
52 146 Stap_Strp_toxin- PFAM
101 127 SAGSUPRFAMLY- PRINTS
116 261 SuperAg_toxin_C_Staph/ Strep- GENE3D
135 148 SAGSUPRFAMLY- PRINTS
137 149 BACTRLTOXIN- PRINTS
139 148 STAPH_STREP_TOXIN_1- PROSITE
140 259 Stap/ Strep_toxin- SSF
154 260 Stap_Strp_tox_C- PFAM
176 199 STAPH_STREP_TOXIN_2- PROSITE
176 192 BACTRLTOXIN- PRINTS
176 189 SAGSUPRFAMLY- PRINTS
189 203 SAGSUPRFAMLY- PRINTS
242 258 SAGSUPRFAMLY- PRINTS

F COPS5
Homo sapiens
GID: 38027922

1 334
40 246 SID
50 191 Mov34/ MPN/ PAD- 1- INTERPRO
57 139 Mov34- 1- INTERPRO
1 334 PTHR10410- PANTHER
1 334 PTHR10410:SF6- PANTHER
50 164 Mov34- PFAM
52 198 SSF102712- SSF
54 191 JAB_MPN- SMART
57 139 Mov34- 1- PRODOM

D CUL3
Homo sapiens
GID: 211971005

1 768
4 221 SID
30 385 Cullin repeat- like- INTERPRO
34 666 Cullin, N- terminal region- INTERPRO
377 676 Cullin homology- INTERPRO
595 632 Winged helix repressor DNA- binding- INTERPRO
662 768 Winged helix repressor DNA- binding- INTERPRO
741 768 Cullin, conserved site- INTERPRO
30 153 G3DSA:1.20.1310.10- GENE3D
30 385 Cullin_repeat- like- SSF
34 666 Cullin- PFAM
159 272 G3DSA:1.20.1310.10- GENE3D
277 381 G3DSA:1.20.1310.10- GENE3D
377 676 Cullin_homology- SSF
381 495 G3DSA:1.20.1310.10- GENE3D
383 635 CULLIN_2- PROFILE
413 563 CULLIN- SMART
462 565 PTHR11932:SF23- PANTHER
462 565 PTHR11932- PANTHER
500 565 G3DSA:4.10.1030.10- GENE3D
588 768 PTHR11932:SF23- PANTHER
588 768 PTHR11932- PANTHER
595 632 Wing_hlx_DNA_bd- GENE3D
662 768 Wing_hlx_DNA_bd- GENE3D
677 768 SSF46785- SSF
741 768 CULLIN_1- PROSITE

D Homolog of jbug (Drosophila mela
Homo sapiens
GID: 281376931

1 193
1 193 SID
1 52 Filamin/ ABP280 repeat- INTERPRO
6 52 Immunoglobulin E- set- INTERPRO
1 52 FILAMIN_REPEAT- PROFILE
6 52 Ig_E- set- SSF

E PAX6 var3
Homo sapiens
GID: 189083680

1 422
67 280 SID
208 229 Coiled domain
4 130 Paired box protein, N- terminal- INTERPRO
5 127 Homeodomain- like- INTERPRO
7 72 Winged helix repressor DNA- binding- INTERPRO
177 273 Homeodomain- related- INTERPRO
197 269 Homeodomain- like- INTERPRO
208 272 Homeobox- INTERPRO
4 128 PAX- SMART
4 128 PAX- PFAM
4 130 PAIRED_2- PROFILE
5 127 Homeodomain_like- SSF
7 72 Wing_hlx_DNA_bd- GENE3D
8 23 PAIREDBOX- PRINTS
13 100 PTHR19418:SF73- PANTHER
13 100 PTHR19418- PANTHER
26 44 PAIREDBOX- PRINTS
38 54 PAIRED_1- PROSITE
46 63 PAIREDBOX- PRINTS
64 81 PAIREDBOX- PRINTS
73 137 Wing_hlx_DNA_bd- GENE3D
177 273 Homeodomain- rel- GENE3D
197 269 Homeodomain_like- SSF
202 381 PTHR19418:SF73- PANTHER
202 381 PTHR19418- PANTHER
208 268 HOMEOBOX_2- PROFILE
210 272 HOX- SMART
211 267 Homeobox- PFAM
213 267 Homeobox- PRODOM
243 266 HOMEOBOX_1- PROSITE
247 257 HOMEOBOX- PRINTS
257 266 HOMEOBOX- PRINTS

E PLEKHH1
Homo sapiens
GID: 110225343

1 1364
51 1364 SID
29 71 Coiled domain
89 124 Coiled domain
139 160 Coiled domain
562 672 Pleckstrin homology- type- INTERPRO
578 674 Pleckstrin- like- INTERPRO
686 795 Pleckstrin homology- type- INTERPRO
687 798 Pleckstrin- like- INTERPRO
832 986 Unconventional myosin/ plant kinesin- like- INTERPRO
993 1333 Band 4.1, N- terminal- INTERPRO
1096 1222 FERM/ acyl- CoA- binding protein, 3- helical- INTERPRO
1 1364 PTHR22903- PANTHER
1 1364 PTHR22903:SF4- PANTHER
562 672 PH_type- GENE3D
576 678 SSF50729- SSF
578 672 PH_DOMAIN- PROFILE
579 674 PH- SMART
579 672 PH- PFAM
682 804 SSF50729- SSF
686 795 PH_type- GENE3D
687 796 PH_DOMAIN- PROFILE
688 796 PH- PFAM
688 798 PH- SMART
832 986 MyTH4- SMART
832 986 MYTH4- PROFILE
874 986 MyTH4- PFAM
993 1230 B41- SMART
997 1333 FERM_3- PROFILE
1093 1225 SSF47031- SSF
1096 1222 ACBP- GENE3D

F RANBP9
Homo sapiens
GID: 39812377

1 729
148 487 SID
147 334 B302, (SPRY)- like- INTERPRO
212 333 SPla/ RYanodine receptor SPRY- INTERPRO
365 397 LisH dimerisation motif- INTERPRO
367 393 LisH dimerisation motif, subgroup- INTERPRO
403 460 CTLH, C- terminal to LisH motif- INTERPRO
615 717 CT11- RanBPM- INTERPRO
147 334 B302_SPRY- PROFILE
174 715 PTHR12864:SF1- PANTHER
174 715 PTHR12864- PANTHER
212 333 SPRY- PFAM
212 333 SPRY- SMART
365 397 LisH- SMART
365 397 LISH- PROFILE
367 393 LisH- PFAM
403 460 CTLH- SMART
403 460 CTLH- PROFILE
615 717 CRA- SMART

E SNAPIN
Homo sapiens
GID: 209870093

1 136
34 136 SID
85 120 Coiled domain
1 133 Snapin- INTERPRO
1 133 Snapin- PIRSF
20 128 PD189673- PRODOM

HYBRiGENiCS

services

DomSight: HBEC_RP1_hgx2542v1 vs. Human Breast Tumor Epithelial Cells_RP1 (05 Nov 2010)

(Bait plasmid(s): hgx2542v1_pB27, hgx2542v1_pB66)

Legend	
bait fragment	*SID: Selected Interaction Domain*
sid fragment	*It is the amino acid sequence shared*
interpro domain	*by all prey fragments matching the*
other domain (pfam, smart, ...)	*same reference protein.*
transmembrane domain	*SIDs have been found in numerous cases*
coiled-coil domain	*to correspond to an identified structural*
signal peptide	*or functional domain.*

legend

	Match	Organism	GID	Length	Domains
D	GenMatch	Homo sapiens	GID: 27753678	1–287	1–273 SID<br>225–247 Transmembrane domain<br>11–12 Coiled domain
D	GenMatch	Homo sapiens	GID: 302313146	1–270	1–148 SID
D	GenMatch	Homo sapiens	GID: 5001538	1–267	1–267 SID<br>20–42 Transmembrane domain<br>52–74 Transmembrane domain<br>91–113 Transmembrane domain<br>155–174 Transmembrane domain<br>1–29 Signal peptide domain
D	GenMatch	Homo sapiens	GID: 83699678	1–141	1–141 SID<br>1–19 Signal peptide domain<br>14–17 Coiled domain<br>49–53 Coiled domain<br>54–55 Coiled domain
D	GenMatch	Homo sapiens	GID: 14091935	1–163	1–163 SID<br>34–56 Transmembrane domain
D	GenMatch	Homo sapiens	GID: 22549655	1–195	1–195 SID<br>82–99 Transmembrane domain<br>138–160 Transmembrane domain<br>172–191 Transmembrane domain
D	GenMatch	Homo sapiens	GID: 23307834	1–142	1–142 SID<br>7–29 Transmembrane domain<br>69–91 Transmembrane domain<br>1–24 Signal peptide domain
D	GenMatch	Homo sapiens	GID: 296785049	1–184	1–184 SID<br>7–119 PTHR12138-PANTHER
D	GenMatch	Homo sapiens	GID: 20087104	1–226	1–226 SID<br>26–48 Transmembrane domain<br>61–83 Transmembrane domain<br>88–107 Transmembrane domain<br>153–175 Transmembrane domain<br>1–17 Signal peptide domain
D	GenMatch	Homo sapiens	GID: 28173064	1–196	1–196 SID<br>70–92 Transmembrane domain<br>159–181 Transmembrane domain<br>1–20 Signal peptide domain
D	GenMatch	Homo sapiens	GID: 22771015	1–231	1–231 SID<br>17–39 Transmembrane domain<br>97–119 Transmembrane domain<br>1–31 Signal peptide domain<br>1–71 PTHR12138-PANTHER
D	GenMatch	Homo sapiens	GID: 7243869	1–121	1–121 SID<br>48–70 Transmembrane domain
