# Supplementary material for: Staphylococcal Enterotoxin O Exhibits Cell Cycle Modulating Activity
Source: Front Microbiol. 2016 Apr 15;7:441. doi: 10.3389/fmicb.2016.00441 (PMC4832122; doi:10.3389/fmicb.2016.00441)
Supplement: Supplementary file 2 [file Data_Sheet_2.PDF]

HYBRiGeNiCS

services

Results Summary

Staphylococcus aureus - SEO
# vs Human Breast Tumor Epithelial Cells_RP1

Fri, Nov 5, 2010 - 10:44 AM

Screen Parameters

Nature	cDNA
Reference Bait Fragment	Staphylococcus aureus - SEO (30-261) ; hgx2542v1
Prey Library	Human Breast Tumor Epithelial Cells_RP1
Vector(s)	pB66 (N-GAL4-bait-C fusion)
Processed Clones	56 (pB66)
Analyzed Interactions	60.9 millions (pB66)
3AT Concentration	0.0 mM (pB66)

Global PBS®

Global PBS (for Interactions represented in the Screen)		Nb	%
A	Very high confidence in the interaction	0	0.0%
B	High confidence in the interaction	0	0.0%
C	Good confidence in the interaction	0	0.0%
D	Moderate confidence in the interaction<br>This category is the most difficult to interpret because it mixes two classes of interactions :<br>- False-positive interactions<br>- Interactions hardly detectable by the Y2H technique (low representation of the mRNA in the library, prey folding, prey toxicity in yeast)	14	73.7%
E	Interactions involving highly connected prey domains, warning of non-specific interaction. The threshold for high connectivity is 10 for screens with Human, Mouse, Drosophila and Arabidopsis and 6 for all other organisms. They can be classified in different categories:<br>- Prey proteins that are known to be highly connected due to their biological function<br>- Proteins with a prey interacting domain that contains a known protein interaction motif or a biochemically promiscuous motif	3	15.8%
F	Experimentally proven technical artifacts	2	10.5%
Non Appliable			
N/A	The PBS is a score that is automatically computed through algorithms and cannot be attributed for the following reasons :<br>- All the fragments of the same reference CDS are antisense<br>- The 5p sequence is missing<br>- All the fragments of the same reference CDS are either all OOF1 or all OOF2<br>- All the fragments of the same reference CDS lie in the 5' or 3' UTR		

HYBRIGENICS CONFIDENTIAL - 672666 - G. Lina

Prey Fragment Analysis

Symbols	Means
✻	The fragment contains the full length CDS
↶	Fragment is fully in 5' UTR
↷	Fragment is fully in 3' UTR
✗	Fragment contains at least one In Frame STOP codon
[NR]	Fragment was found to be non relevant (poor quality, high N density)
IF<br>OOF1<br>OOF2	With regard to the theoretical frame of each corresponding CDS (GeneBank),fragments are cloned in frame (IF) if they are in the same frame as Gal4AD.In general, polypeptides synthesized from OOF fragments are not considered of biological interest, unless found together with another frame. However, some of the proteins expressed from an OOF fragment can be translated in the correct frame,due to the existence of natural frame-shift events during translation in yeast
??	Unidentified frame when :<br>- The clone sequence is antisense<br>- The 5p sequence is missing
N	Antisense
Start...Stop	Position of the 5p and 3p prey fragment ends, relative to the position of the ATG start codon (A=0)

Clone Name	Type Seq	Gene Name (Best Match)	Start..Stop (nt)	Frame	Sense	%Id 5p	%Id 3p	PBS
pB66_A-14	5p/3p	Homo sapiens - COPS5	39..918	IF		90.1	96.9	F
pB66_A-48	5p/3p	Homo sapiens - COPS5	39..918	IF		95.0	93.9	F
pB66_A-13	5p/3p	Homo sapiens - COPS5	39..918	IF		93.6	95.4	F
pB66_A-12	5p/3p	Homo sapiens - COPS5	39..918	IF		96.2	96.2	F
pB66_A-54	5p/3p	Homo sapiens - COPS5	96..738	IF		84.7	99.5	F
pB66_A-5	5p/3p	Homo sapiens - COPS5	111..916	IF		95.0	97.8	F
pB66_A-50	5p/3p	Homo sapiens - COPS5	111..825	IF		97.5	96.9	F
pB66_A-35	5p	Homo sapiens - COPS5	111	IF		86.6		F
pB66_A-28	5p/3p	Homo sapiens - COPS5	111..916	IF		94.1	94.3	F
pB66_A-6	5p/3p	Homo sapiens - COPS5	111..825	IF		97.8	96.6	F
pB66_A-59	5p/3p	Homo sapiens - COPS5	111..916	IF		94.8	97.4	F
pB66_A-27	5p/3p	Homo sapiens - COPS5	111..916	IF		95.8	95.4	F
pB66_A-18	5p	Homo sapiens - COPS5	111	IF		76.3		F
pB66_A-37	5p/3p	Homo sapiens - COPS5	111..916	IF		94.8	95.2	F
pB66_A-39	5p/3p	Homo sapiens - COPS5	117..815	IF		96.7	97.8	F
pB66_A-32	5p/3p	Homo sapiens - COPS5	117..815	IF		95.7	97.9	F
pB66_A-62	5p	Homo sapiens - COPS5	117	IF		96.9		F
pB66_A-69	5p/3p	Homo sapiens - COPS5	117..815	IF		98.1	99.3	F
pB66_A-56	5p/3p	Homo sapiens - CUL3	9..665	IF		98.8	98.3	D
pB66_A-9	5p	Homo sapiens - DKFZP566N034	1053 ↷	IF		71.8		N/A
pB66_A-23	5p/3p	Homo sapiens - FLJ10211	2142..2868 ↷ ✗	IF		95.7	96.4	N/A
pB66_A-20	5p/3p	Homo sapiens - FUT9	3321..4068 ↷ ✗	IF		ALU 93.8	ALU 96.2	N/A
pB66_A-21	5p/3p	Homo sapiens - GK5	5931..6606 ↷ ✗	IF		ALU 96.7	ALU 96.9	N/A
pB66_A-10	5p	Homo sapiens - Homolog of jbug (Drosophila melanogaster) GID: 281376931	-1	IF		100.0		D
pB66_A-47	5p/3p	Homo sapiens - MAPK6	1529..949	??	N	98.5	99.5	N/A
pB66_A-3	5p/3p	Homo sapiens - PAX6 var3	198..840	IF		99.5	98.8	E
pB66_A-46	5p/3p	Homo sapiens - PAX6 var3	198..840	IF		96.4	97.5	E

Clone Name	Type Seq	Gene Name (Best Match)	Start..Stop (nt)	Frame	Sense	%Id 5p	%Id 3p	PBS
pB66_A-43	5p	Homo sapiens - PLEKHH1	150	IF		98.7		E
pB66_A-36	5p/3p	Homo sapiens - RANBP9	372..1498	IF		98.3	96.5	F
pB66_A-51	5p/3p	Homo sapiens - RANBP9	372..1498	IF		96.5	95.2	F
pB66_A-64	5p/3p	Homo sapiens - RANBP9	396..1461	IF		93.1	96.7	F
pB66_A-58	5p/3p	Homo sapiens - RANBP9	405..1509	IF		94.5	97.0	F
pB66_A-38	5p/3p	Homo sapiens - RANBP9	441..1516	IF		97.6	93.7	F
pB66_A-4	5p/3p	Homo sapiens - SFRS18	962..466	??	N	99.6	99.8	N/A
pB66_A-65	5p/3p	Homo sapiens - SFRS18	962..466	??	N	99.6	99.8	N/A
pB66_A-63	5p/3p	Homo sapiens - SFRS18	962..466	??	N	98.0	98.6	N/A
pB66_A-30	5p/3p	Homo sapiens - SFRS18	962..466	??	N	99.4	98.4	N/A
pB66_A-40	5p	Homo sapiens - SFRS18	962	??	N	93.5		N/A
pB66_A-49	5p/3p	Homo sapiens - SFRS18	962..466	??	N	99.8	99.8	N/A
pB66_A-2	5p	Homo sapiens - SFRS18	962	??	N	91.8		N/A
pB66_A-22	5p/3p	Homo sapiens - SNAPIN	99..438	IF		100.0	100.0	E
pB66_A-55	5p/3p	Homo sapiens - TMEM163	1053..1739	IF		97.5	98.1	N/A
pB66_A-45	5p/3p	Homo sapiens - GenMatch GID: 27753678	-1..820	IF		ALU 85.2	ALU 60.9	D
pB66_A-24	5p/3p	Homo sapiens - GenMatch GID: 302313146	-1..446	IF		100.0	59.3	D
pB66_A-16	5p/3p	Homo sapiens - GenMatch GID: 5001538	-1..844	IF		100.0	93.8	D
pB66_A-15	5p/3p	Homo sapiens - GenMatch GID: 83699678	-1..659	IF		ALU 68.4	63.3	D
pB66_A-1	5p	Homo sapiens - GenMatch GID: 14091935	-1	IF		100.0		D
pB66_A-66	5p/3p	Homo sapiens - GenMatch GID: 22549655	-1..586	IF		100.0	99.3	D
pB66_A-61	5p	Homo sapiens - GenMatch GID: 22549655	-1	IF		89.7		D
pB66_A-60	5p/3p	Homo sapiens - GenMatch GID: 23307834	-1..718	IF		ALU 97.2	ALU 99.8	D
pB66_A-19	5p/3p	Homo sapiens - GenMatch GID: 296785049	-1..552	IF		ALU 99.1	ALU 99.8	D
pB66_A-42	5p/3p	Homo sapiens - GenMatch GID: 296785049	-1..552	IF		ALU 99.6	ALU 100.0	D
pB66_A-29	5p/3p	Homo sapiens - GenMatch GID: 20087104	-1..842	IF		94.9	ALU 89.5	D
pB66_A-11	5p/3p	Homo sapiens - GenMatch GID: 28173064	-1..588	IF		ALU 99.5	ALU 99.7	D
pB66_A-8	5p/3p	Homo sapiens - GenMatch GID: 22771015	-1..696	IF		ALU 99.4	ALU 98.3	D
pB66_A-7	5p/3p	Homo sapiens - GenMatch GID: 7243869	-1	IF		99.2	72.2	D
