# Supplementary figures and images for: Staphylococcal Enterotoxin O Exhibits Cell Cycle Modulating Activity
Source: Front Microbiol. 2016 Apr 15;7:441. doi: 10.3389/fmicb.2016.00441 (PMC4832122; doi:10.3389/fmicb.2016.00441)

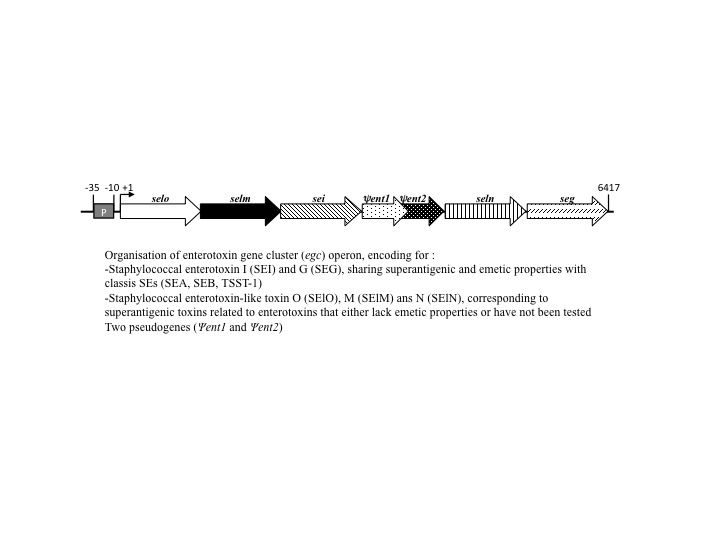

Supplement: Supplementary file 3 [file Image_1.JPEG]
